# High levels of pro-inflammatory SARS-CoV-2-specific biomarkers revealed by *in vitro* whole blood cytokine release assay (CRA) in recovered and long-COVID-19 patients

**DOI:** 10.1371/journal.pone.0283983

**Published:** 2023-04-05

**Authors:** Shayane Martins Rodrigues Gomes, Andréia Carolinne de Souza Brito, Wânia Ferraz Pereira Manfro, Marcelo Ribeiro-Alves, Roberto Stefan de Almeida Ribeiro, Mariana Soares da Cal, Vinicius da Cunha Lisboa, Daniel Paiva Barros de Abreu, Leda dos Reis Castilho, Luís Cristóvão de Moares Sobrino Porto, Thiago Thomáz Mafort, Agnaldo José Lopes, Silvia Amaral Gonçalves da Silva, Patrícia Maria Lourenço Dutra, Luciana Silva Rodrigues

**Affiliations:** 1 Department of Microbiology, Parasitology and Immunology, Medical Science Faculty (FCM), Rio de Janeiro State University (UERJ), Rio de Janeiro, RJ, Brazil; 2 Laboratory of Clinical Research on STD/AIDS, National Institute of Infectology Evandro Chagas, Oswaldo Cruz Foundation (FIOCRUZ), Rio de Janeiro, RJ, Brazil; 3 Laboratory of Immunopathology, Department of General Pathology and Laboratories, FCM, UERJ, Rio de Janeiro, RJ, Brazil; 4 Pulmonary and Tisiology Department, Pedro Ernesto University Hospital (HUPE), Policlínica Piquet Carneiro (PPC)/UERJ, Rio de Janeiro, RJ, Brazil; 5 Chemical Engineering Program, Cell Culture Engineering Lab (COPPE), Federal University of Rio de Janeiro (UFRJ), Rio de Janeiro, RJ, Brazil; 6 Histocompatibility and Cryopreservation Laboratory, Department of Histology and Embryology Institute of Biology Roberto Alcantara Gomes, UERJ, Rio de Janeiro, RJ, Brazil; Instituto Butantan, BRAZIL

## Abstract

**Background:**

Cytokines induced by SARS-CoV-2 infection play a crucial role in the pathophysiology of COVID-19 and hyperinflammatory responses have been associated with poor clinical outcomes, with progression to severe conditions or long-term subacute complications named as long-COVID-19.

**Methods:**

In this cross-sectional study, we aimed to evaluate a set of antigen-specific inflammatory cytokines in blood from recovered COVID-19 individuals or who suffered a post-acute phase of SARS-CoV-2 infection compared to healthy individuals with no history of COVID-19 exposition or infection. Interferon-gamma (IFN-γ), IFN-γ-induced protein 10 (IP-10), tumor necrosis factor (TNF), IL-1β, IL-2, IL-4, IL-6, IL-8, IL-10, IL-12, and IL-17A were quantified by multiplex cytometric bead assay and enzyme-linked immunosorbent assay after stimulation of whole blood with recombinant Spike protein from SARS-CoV-2. Additionally, all participants have evaluated for anti-(S) protein-specific IgG antibodies. Clinical specimens were collected within two months of COVID-19 diagnosis.

**Results:**

A total of 47 individuals were enrolled in the study, a median age of 43 years (IQR = 14.5), grouped into healthy individuals with no history of infection or exposure to SARS-CoV-2 (unexposed group; N = 21); and patients from the Health Complex of the Rio de Janeiro State University (UERJ), Brazil, who were SARS-CoV-2 positive by RT-PCR (COVID-19 group)–categorized as recovered COVID-19 (N = 11) or long-COVID-19 (N = 15). All COVID-19 patients presented at least one signal or symptom during the first two weeks of infection. Six patients were hospitalized and required invasive mechanical ventilation. Our results showed that COVID-19 patients had significantly higher levels of IFN-γ, TNF, IL-1β, IL-2, IL-6, IL-8, and IP-10 than the unexposed group. The long-COVID-19 group has presented significantly high levels of IL-1β and IL-6 compared to unexposed individuals, but not from recovered COVID-19. A principal-component analysis demonstrated 84.3% of the total variance of inflammatory-SARS-CoV-2 response in the first two components, and it was possible to stratify IL-6, TNF, IL-1β, IL-10, and IL-2 as the top-five cytokines which are candidates to discriminate COVID-19 group (including long-COVID-19 subgroup) and healthy unexposed individuals.

**Conclusion:**

We revealed important S protein-specific differential biomarkers in individuals affected by COVID-19, bringing new insights into the inflammatory status or SARS-CoV-2 exposition determination.

## Introduction

The novel coronavirus SARS-CoV-2 (Severe Acute Respiratory Syndrome Coronavirus 2), which emerged in Wuhan-China in December 2019, has caused morbidity and mortality on an unprecedented scale, reaching a dramatic hallmark of almost 629 million confirmed cases and more than 6.5 million deaths worldwide by November 2022, as reported by the World Health Organization (WHO) [1]. At the same time, many patients who have recovered from acute COVID-19 infection have persistent symptoms (termed long-COVID-19), leading to significant disability and impairment of their activities of daily living, and are likely to continue to require massive medical assistance in the future [2]. It is still unclear to what extent the evolution of the virus and the emergence of new variants can increase pathogenesis, escape immunity generated by previous infection or vaccination, or induce more severe disease and its sequelae [3].

Once a person is infected, the innate immune system uses a variety of pattern recognition receptors (PRRs) to detect the presence of RNA viruses, such as Toll-like receptors or other cytosolic RNA sensors, which trigger a broad signaling cascade in effector cells to mediate viral clearance. Interferons (IFNs), a class of cytokines grouped in three families (type I, II, and III), work in a synergistic manner to provide the universal antiviral immune response and are crucial for the control of viral replication and dissemination. The production of types I (alpha/beta, mainly) and III (lambda) IFNs by a variety of cell types, and consequently activation of natural killers (NK), for example, can eliminate viruses and infected cells. However, SARS-CoV-2 can evade these early defense mechanisms by suppression of IFN-I and IFN-III activities and cellular function, promoting a dysfunctional immune response and consequently, the establishment of infection [4]. Additionally, type II IFN (gamma; IFN-γ), produced by NK cells and T lymphocytes, contributes to the establishment of an antiviral state and regulating cell-mediated responses, such as macrophage activation and enhancement of antigens presentation [5–7].

In severe cases of COVID-19, progression to acute respiratory distress syndrome (ARDS) is often mediated by alteration of the innate immune system that leads to an overproduction of pro-inflammatory cytokines (named as “cytokine storm”) [8, 9]. In parallel, the adaptive immune response, involving humoral and cellular counterparts, is crucial for infection control and memory immunity generation. In that sense, antibody response to SARS-CoV-2 infection has been widely described [10, 11]. On the other hand, some studies have shown that cellular immunity seems to be more constant and lasting in individuals infected with SARS-CoV-2, even if asymptomatic [12, 13]. Evidence suggests that dysregulated inflammation with exacerbated production of inflammatory cytokines, such as IL-1β, IL-6, and IL-8, plays a significant role in patients with severe COVID-19 [14–16]. The most severe clinical conditions seem to be characterized by a slow decline in viral load and increased levels of TNF and IFN-γ [14]. Furthermore, a multi-omic assessment in progressive COVID-19 patients showed that the desynchronized adaptive and innate immune response might lead to delayed virus clearance [17].

Although COVID-19 predominantly affects the lungs, it is a multisystemic disease that often results in significant sequelae or even death [18–21]. Long-COVID-19, or post-COVID-19 syndrome, is characterized by clinical manifestations during or after a SARS-CoV-2 infection that continues for more than 12 weeks and is not explained by an alternative diagnosis [22]. These long-COVID-19 sequelae are not yet fully understood, but there is already evidence that SARS-CoV-2 can deteriorate lung function, negatively impact cardiovascular, neurological, and muscle systems, impair physical function, and beyond the reduced quality of life and cause significant emotional damage [23, 24]. A spectrum of clinical manifestations, including dyspnea, chest pain, palpitations, general fatigue, decreased exercise capacity, neurocognitive difficulties, muscle pain, muscle weakness, myocarditis, kidney failure, depression, anxiety, and other mental health conditions, have been reported among the survivors of COVID-19 [25, 26]. However, little is known about the cellular immunity involved in immunopathology of long-COVID-19, and the causes or mechanisms which may be associated with reduced functionality in individuals with this kind of outcome are yet to be investigated.

In this scenario, the present work aimed to investigate the inflammatory profile of COVID-19 patients who recovered or experienced the long-COVID-19 compared to those without a history of infection or exposition to SARS-CoV-2, accessing the cellular antigen-specific immune response in a cytokine release assay (CRA) using unfractionated peripheral blood.

## Methods

### Study design and participants

The study was carried out following the principles of the Declaration of Helsinki. The Ethical Committee of the Rio de Janeiro State University approved the study (#4.649.707), which was carried out between November 2020 and May 2021. An informed consent form was required to enroll patients and control physicians.

This cross-sectional study was performed on subjects aged ≥ 18 years with different SARS-CoV-2 exposure or infection status in a pre-COVID-19 vaccine period. These subjects were being followed up at the Pedro Ernesto University Hospital (HUPE)/Piquet Carneiro Polyclinic (PPC) of the Health Complex of the Rio de Janeiro State University (UERJ), Rio de Janeiro, RJ, Brazil. All COVID-19 cases were defined by the positivity of nasopharyngeal swabs for SARS-CoV-2 by reverse-transcription polymerase chain reaction (RT-PCR) and classified as mild, moderate, and severe, according to the WHO [27]. The Control group consisted of healthy volunteers who had been in home isolation during the early period of COVID-19 emergence and did not refer any history of suspicious signal/symptoms or contact with COVID-19 cases, at least until sample collection. At the enrollment of the study, all participants had negative results for SARS-CoV-2 RT-PCR, and none of them had been vaccinated against SARS-CoV-2. All blood collections were performed up to two months after the diagnosis of COVID-19. A total of 47 participants were enrolled in this study and grouped as follows: i) COVID-19 patients (N = 26) and ii) unexposed individuals (N = 21). The COVID-19 group was subdivided into 11 recovered COVID-19 participants with full recovery within 14 days of COVID-19 diagnosis; and 15 Long-COVID-19 patients, characterized by persistent manifestations and symptoms consistent with COVID-19 from > 4 weeks [25]. Clinical data, including sociodemographic and anthropometric information and the patient’s health history and clinical evolution, were acquired from the medical records.

### Recombinant SARS-CoV-2 Spike protein

Recombinant SARS-CoV-2 spike protein (S protein) was produced in HEK293 cells as previously described by Alvim and collaborators [28]. Briefly, the spike protein’s soluble ectodomain (amino acids 1–1208 Wuhan strain) was expressed as a trimer stabilized in the pre-fusion conformation. The trimeric spike protein was purified from cell culture supernatants by affinity chromatography. Protein concentration, purity, and identity in the eluted fractions were confirmed by NanoDrop (Thermo Fisher Scientific™), silver-stained SDS-PAGE, and Western blot analyses, respectively. Endotoxin level in the purified protein preparations was detected using the Pierce™ Chromogenic Endotoxin Quant Kit (Thermo Fisher Scientific™). Endotoxin levels did not exceed 0.0052 endotoxin units per μg of protein (0.0052 EU/μg).

### Sample collection and stimulation

Peripheral blood samples were collected in vacuum tubes with sodium heparin (BD Vacutainer, Becton Drive, Franklin Lakes, USA). One milliliter of whole blood was deposited in sterile round-bottomed polystyrene test tubes (Corning Science Mexico S.A. de C.V., Reynosa, Mexico) and stimulated or not as follows: i) negative control tube: no stimulation; ii) Recombinant SARS-CoV-2 Spike protein at 5 μg/mL; or iii) phytohemagglutinin-M (PHA; Roche Diagnostics GmbH, Mannheim, Germany) at 5 μg/mL, as the positive control (mitogen). After adding the stimuli, whole blood was incubated for 24 h in a humidified atmosphere at 37°C (5% CO_2_). Supernatants were harvested/processed by centrifugation at 3000 g for 15 min and stored at -80°C until cytokine measurements. Recombinant SARS-CoV-2 Spike protein was filtered and diluted in sterile endotoxin-free PBS. PHA was reconstituted in double-distilled water, according to manufacturer recommendation, and diluted in sterile endotoxin-free PBS.

### Cytokine release assay (CRA)

Cytokine levels in stimulated whole blood supernatants were measured using the following commercial kits: i) Human Th1/Th2/Th17 and Human Inflammatory Cytokine Kits (BD Bioscience, San Jose, CA, USA) based on the principle of multiplex cytometric bead array (CBA) technology for detection of IL-1β, IL-2, IL-4, IL-6, IL-8, IL-10, IL-12, TNF, IFN-γ, and IL-17A. Briefly, undiluted samples were incubated with capture beads labeled with distinct fluorescence intensity (allophycocyanin; APC) conjugated with specific antibodies for cytokines and fluorescent detection antibody (phycoerythrin; PE) for around 3 hours at room temperature and protected from light. All unbound antibodies were washed, and samples were acquired on a BD fluorescence-activated cell sorting (FACS) analyzer FACSCanto II. Cytokine standard curves ranged from 0–5,000 pg/mL; ii) IP-10 was assessed by enzyme-linked immunosorbent assay (ELISA) sandwich using human CXCL-10/IP-10 DuoSet ELISA (R&D Systems Inc, MN, USA) following the manufacturer’s instruction. The range of these assays was 31.3–10,000 pg/mL; and iii) Human IFN-γ ELISA from Quanti-FERON kit (Qiagen). The range of this last assay was 0.065 IU/mL to 10.0 IU/mL. Cytokine-specific levels were calculated by subtracting the value obtained from the negative control tube. A positive response in the *COVID-19-Cellular Immunity test* was determined by IFN-γ (Quanti-FERON kit)-specific levels in response to SARS-Cov-2 S protein antigen minus negative control tube at 0.562 IU/mL cut-off point and simultaneous response to mitogen (PHA minus negative control) up to a cut-off point of 3.47 IU/mL.

### Anti-SARS-CoV-2 (Spike protein) IgG serology

High-binding ELISA plates (Corning, #3590) were coated with 50 μL of recombinant SARS-CoV-2 Spike protein in carbonate-bicarbonate buffer (pH 9.4) at a concentration of 4μg/mL (200 ng/well) and incubated overnight at room temperature (RT), as described by Alvim *et al*. [28]. The coating solution was removed, and the blocking solution (2% Skim milk in phosphate-buffered saline-PBS, 150 μL per well) was added to the plates and incubated at RT for 1 hour. The blocking solution was removed, and 50 μL of 3-step, 5-fold serial dilutions of patient sera (1:40, 1:200, 1:1000) were added and incubated at RT for 1.5 hours. The plates were washed with 250 μL PBS plus 0.05% Tween-20 three times with a final aspiration step using a plate washer (Immuno washer 1575, Bio-Rad). A goat anti-human IgG (H+L) HRP-conjugate (Invitrogen, #A18805, 1:10,000, 50 μL/well) was added, followed by incubation for 1.5 hours at RT. The plates were washed as previously described, and TMB Chromogen Solution (Life Technologies, #002023, 50 μL/well) was added, followed by incubation for 15 minutes at RT. The reaction was stopped by adding 50 μL/well of 1 N HCl, and the plates were read in a microplate reader (iMark™, Bio-Rad) at 450 nm. All 450 nm data were corrected by subtracting the respective O.D. (Optical Density) up to 655 nm for background compensation. For quality control purposes, a given RT-PCR positive sample was serially diluted in the same way (1:40, 1:200, 1:1000) and added in duplicate to each plate. Results were expressed as the ratio of O.D. (450 to 655 nm) of samples at the 1:40 dilution divided by the cut-off, which was defined as the O.D. mean of 12 pre-pandemic negative controls added to the same ELISA plate plus three times the standard deviation of a separate plate run with 92 pre-pandemic controls [28]. An O.D. ratio below 0.9 was considered a negative sample, an O.D. ratio above 1.8 was considered a positive sample, and an O.D. ratio between them was considered undetermined. Additionally, the absorbance summation (O.D. Sum) was also calculated by adding the O.D. values of all dilutions of each sample to obtain one data point per sample, facilitating data comparison as proposed by Hartman *et al*. [29].

### Statistical analysis

For the description of the population included in the study according to their sociodemographic and clinical characteristics among individuals either unexposed to SARS-CoV-2 or with COVID-19, for continuous numerical variables the nonparametric Mann-Whitney tests were used, while for comparison of the relative frequencies of the different levels of nominal/categorical variables Fisher’s exact tests were used. Besides, Pearson’s rank correlation coefficient analyses were estimated for continuous numerical variables. In comparing the levels of log-transformed expression of cytokine S protein-specific production between individuals belonging to groups of unexposed to SARS-CoV-2, with COVID-19, recovered from COVID-19, or with long-COVID-19, the expected mean marginal values were obtained by multiple linear regression (log-linear) models of fixed effects, including the main group effects. The main effect was corrected for confounding variables (e.g., age, BMI, and sex) in the systematic component of the models. Marginal mean values and their 95% confidence intervals were then estimated by keeping all confounders in the multiple linear models at their mean values or equal proportions. Contrasts were constructed from these estimated marginal mean values. Pairwise p-values were corrected for the number of comparisons (3-groups only) using the Holm-Sidak method. For the adjusted models, graphical analyses of the residuals were performed to confirm their randomness. Finally, a multivariate principal-component analysis (PCA) was performed for the log-transformed expression data to visualize the distribution of sample individuals in two-dimensional (2D) spaces. Before that, we proceeded with a greedy iterative “highly predictive” variables selection until finding an acceptable level of 0.9 for the standardized Cronbach’s coefficient alpha. The proportion of explained variation was calculated after each eigenvalue. The cumulative percentage explained is obtained by adding the successive proportions of variation explained to obtain the running total. The contributions (in percentage) of the variables to the principal components were calculated as (var. cos^2^ × 100)/(total cos^2^ of the component), where cos^2^ indicates square cosine or squared coordinates. Accordingly, the contributions (in percentage) of individuals to the principal components were calculated as (ind. cos^2^ × 100)/(total cos^2^ of the component). Ellipses of the quantiles 66% of the normal distribution adjusted to the individuals of the different interest groups in these new dimensional spaces are presented. A P-value ≤ 0.05 was used as the significance level in the analysis. All analyses were performed usingR software version 4.1.2 [30], and packages ‘base’ for descriptive and correlation analyses, ‘lme4’ [31] and ‘emmeans’ [32] for model inferences, ‘FactoMineR’ [33]and ‘factoextra’ [34] for PCA and its graphic representation, and their dependencies.

## Results

### Characteristics of the study population

**Fig 1:**
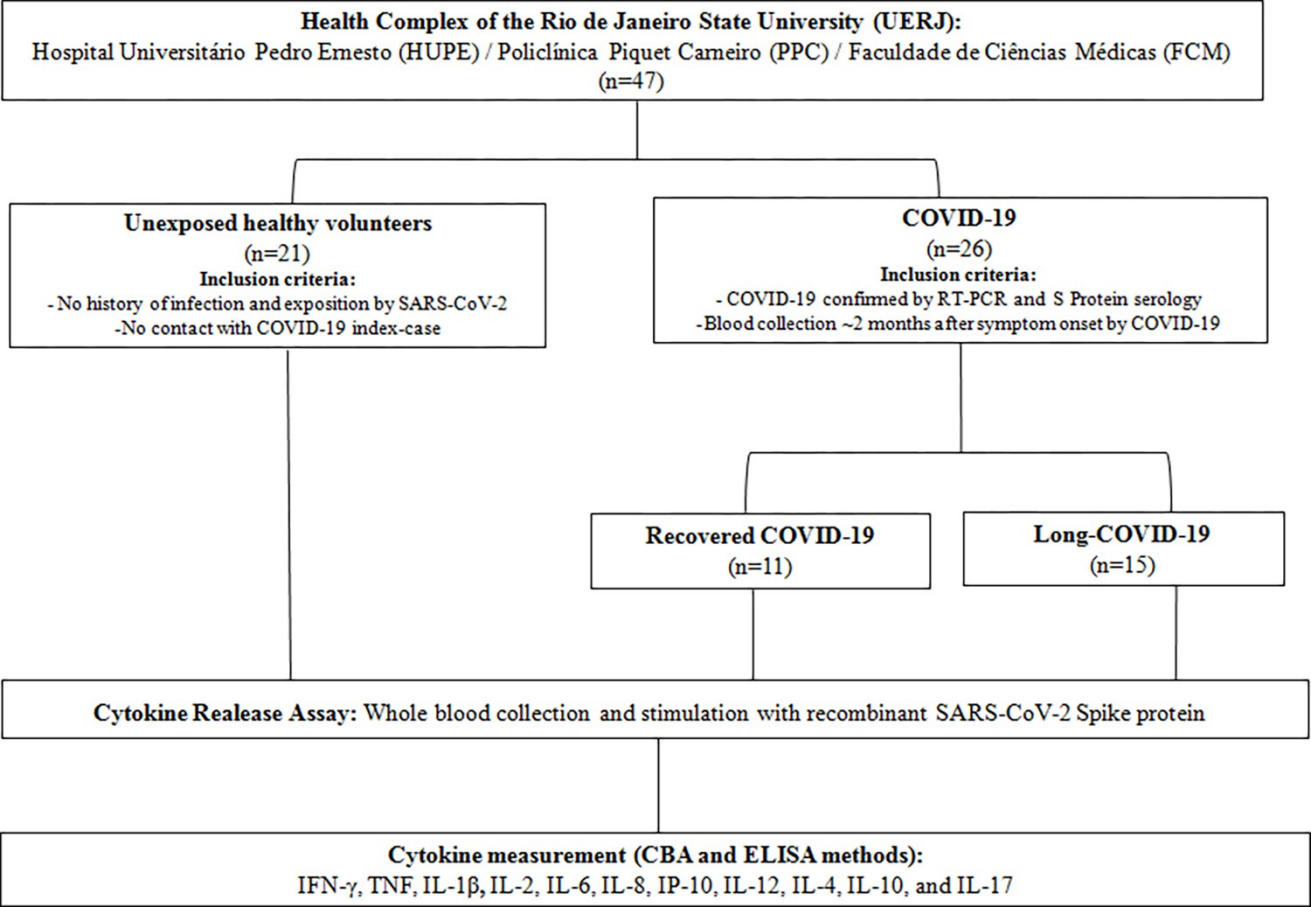
Study design. Flow chart showing recruitment sites, eligibility criteria for selecting volunteers, and methodologies used for sample processing.

The study population consisted of 47 participants, including 21 healthy individuals with no history of exposition or suspected COVID-19 infection who have been quarantined since the WHO declared the pandemic; and 26 individuals who were confirmed COVID-19 cases according to the criteria previously described. Subsequently, the group called COVID-19 was subdivided: i) recovered COVID-19, composed of 11 (42.3%) individuals who fully recovered within 14 days of diagnosis, and ii) long-COVID-19, composed of 15 individuals (57.6%) with persistent clinical manifestations or symptoms even after two months of COVID-19 diagnosis **(Fig 1)**. As shown in **Table 1**, there were no differences in the age of the population studied. However, we observed that the COVID-19 group had a significantly higher body index mass (BMI) and presence of comorbidities than the unexposed control group. The most prevalent signs and/or symptoms of COVID-19 at diagnosis were fever (80.8%), loss of smell (69.2%), headache (57.7%), and cough (57.7%). The degree of severity for COVID-19 was 76.9% for mild cases, 3.8% for moderate cases, and 19.2% for severe cases, representing 23.1% of hospitalization, of which 7.7% required mechanical ventilation.

**Table 1:** Sociodemographic and clinical characteristics of the study population.

Characteristics	Unexposed (n = 21)	COVID-19 (n = 26)	*P-*value
**Demographics**			
Age, yr (IQR)	41 (13)	47.5 (19.5)	0.261
Male, n (%)	7 (33.3)	11 (42.3)	0.743
BMI (IQR)	23.73 (4.83)	31.04 (8.96)	0.001
**Comorbidities, n (%)**			
Obesity	2 (9.5)	14 (53.8)	0.004
Diabetes	1 (4.8)	7 (26.9)	0.105
Arterial hypertension	0 (0)	11 (42.3)	0.002
Others	0 (0)	6 (23.1)	0.05
**Signs and symptoms** ^**a**^ **, n (%)**			
Tiredness	N/A	15 (57.7)	
Fever	N/A	21 (80.8)	
Muscle aches	N/A	9 (34.6)	
Weakness	N/A	9 (34.6)	
Headache	N/A	15 (57.7)	
Anosmia	N/A	18 (69.2)	
Ageusia	N/A	12 (46.2)	
Dyspnea	N/A	8 (30.8)	
Cough	N/A	15 (57.7)	
Diarrhea	N/A	11 (42.3)	
**Severity, n (%)**			
Mild	N/A	20 (76.9)	
Moderate	N/A	1 (3.8)	
Severe	N/A	5 (19.2)	
Hospitalization	N/A	6 (23.1)	
Mechanical ventilation	N/A	2 (7.7)	
**Long-COVID-19, n (%)**	N/A	15 (57.7)	
**Signs and symptom****s**^**b**^**, n = 15/26 (%)**			
Tireness	N/A	11/15 (73.3)	
Cough	N/A	10/15 (66.6)	
Difficulty breathing	N/A	6/15 (40.0)	
Ageusia	N/A	4/15 (26.6)	
Anosmia	N/A	2/15 (13.3)	
Headache	N/A	2/15 (13.3)	
Body aches	N/A	2/15 (13.3)	
Coryza	N/A	1/15 (6.6)	
Chest pain	N/A	1/15 (6.6)	

Yr, year; IQR, interquartile range; BMI, body mass index; N/A, not applicated. Values are numbers of the total population unless otherwise stated. Values in parentheses are percentages from each group.

^a^Signs and symptoms reported at diagnosis of COVID-19.

^b^Signs and symptoms reported in the long-COVID-19 subgroup by the time of blood collection.

P-values obtained by Fisher’s exact tests.

### Cellular and humoral immunity against COVID-19 in the study population

**Fig 2:**
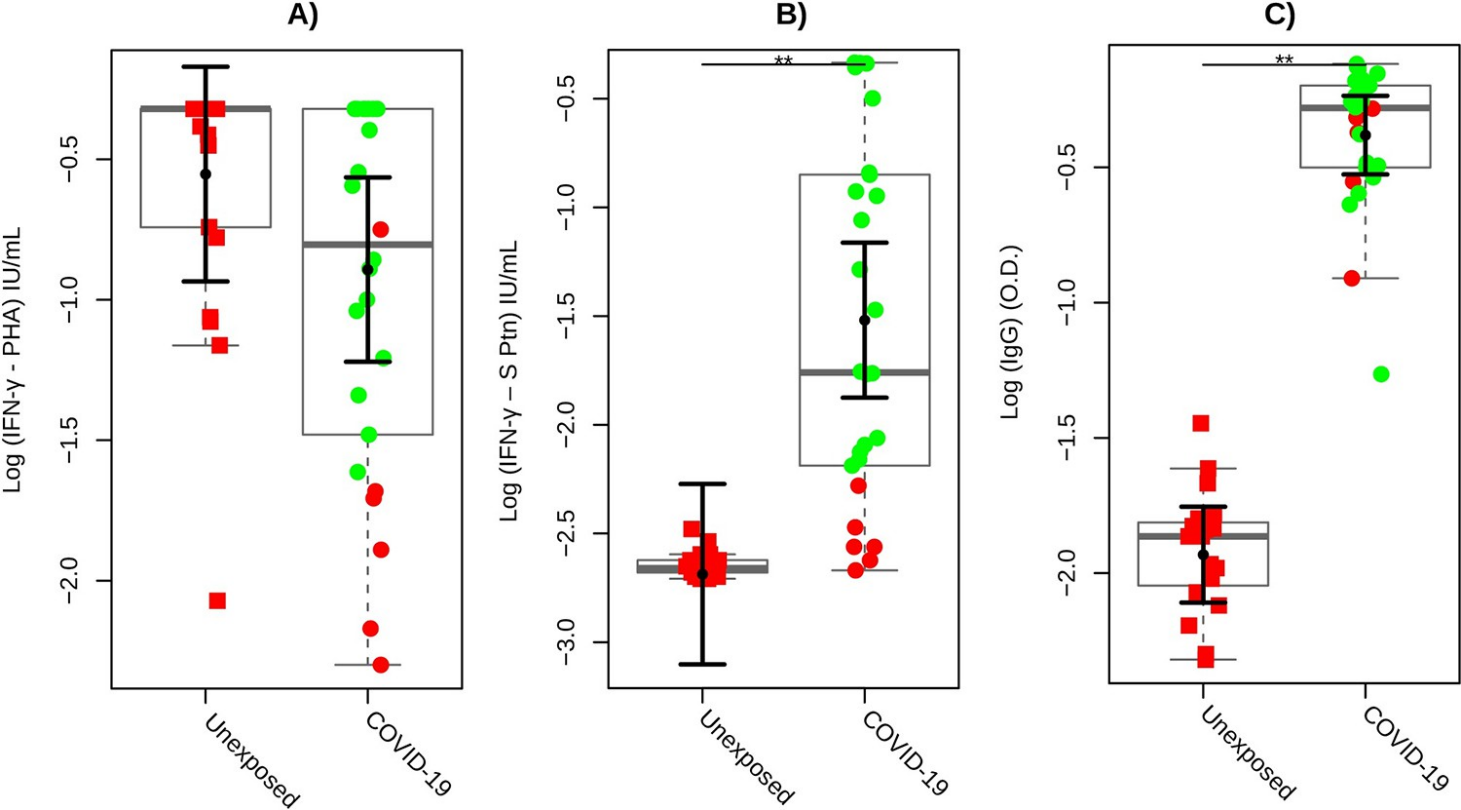
COVID-19 cellular response test. **(A, B)** Mean marginal IFN-γ production to assess cytokine production in response to PHA and SARS-CoV-2 S Protein, respectively, in whole blood *COVID-19-cellular immunity test* among the study population. **(C)** Anti-S protein IgG serology of healthy unexposed, and confirmed volunteers for COVID-19. Green circles indicate positive individuals (responders) for the cellular immune response, while red solid circles indicate those who were negative (non-responders). Solid squares represent individuals in the unexposed group, solid circles represent individuals in the COVID-19 group, and the box plots represent the interquartile range and the sample median (solid gray centerline). The larger black solid circles and central bars represent the fitted means estimated by the linear model, and the marginal mean values and their upper 95% confidence intervals were estimated for both unexposed individuals and for COVID-19 controlled for sex, BMI, and age for IFN-γ and PHA (mitogen) after stimulation with a specific biomarker for SARS-CoV-2 S protein. **, P<0.01.

Firstly, we asked whether antigen-specific interferon-gamma (IFN-γ) production could be accessed as a biomarker of cellular immunity against SARS-CoV-2 in our study population. Whole blood from individuals with different COVID-19 exposition or infection status was stimulated with recombinant SARS-CoV-2 S protein as antigen: unexposed and COVID-19 groups. Peripheral blood was collected from COVID-19 patients within around two months of diagnosis, considered a subacute phase of the disease. As seen in **Fig 2A**, there was no significant difference in IFN-γ levels between the unexposed and COVID-19 groups when mitogen response (positive control) was evaluated (P = 0.09). Interestingly, significantly high levels of IFN-γ-S protein-specific were produced by the COVID-19 group compared to healthy individuals **(Fig 2B**; P<0.01**)**. In parallel, the humoral response against SARS-CoV-2 was accessed by anti-S protein IgG serology. All subjects (26/26) in the COVID-19 group had a positive test, and we observed that this group showed significantly higher antibody titers of anti-S protein IgG than those in the control group **(Fig 2C**; P<0.01**)**, which three individuals (3/21, 14.2%) had undetermined anti-S protein IgG serology and two (2/21, 9.5%) were positive, even though showing borderline results (2.0–2.5 O.D. ratio).

In the next step, we developed a COVID-19-cellular immunity test based on the ability of IFN-γ-S protein-specific levels to discriminate between unexposed healthy and COVID-19 groups by whole blood *in vitro* stimulation assay. Considering the upper 95% CI of IFN-γ SARS-CoV-2 S protein-specific production among unexposed individuals, it was established a cut-off_IFN-γ_ point at 0.562 IU/mL and considering the lower 95% CI of PHA production among COVID-19 individuals, it was established a cut-off_PHA_ at 3.47 IU/mL. Thus, we defined the following possible outcomes: (1) undetermined, such as those with PHA production in whole blood lower than cut-off_PHA_; (2) non-responders or negatives, such as those with PHA production in whole blood higher than cut-off_PHA_ and IFN-γ SARS-CoV-2- S protein-specific production in whole blood lower than cut-off_IFN-γ_; and (3) responders or positives, such as those with PHA production in whole blood higher than cut-off_PHA,_ and IFN-γ SARS-CoV-2- S protein-specific production in whole blood higher or equal than cut-off_IFN-γ_. There were no indeterminate results for the study population.

Further exploring **Fig 2**, representative colors show that the COVID-19 group presented 76.92% (20/26) of positive cellular response (green solid circles) accompanied by high levels of IFN-γ in response to SARS-CoV-2 S protein. In contrast, the unexposed healthy group did not present a positive cellular response (red solid circles). Only 23.07% (06/26) of the volunteers in the COVID-19 group did not show a positive cellular response and similar levels of IFN-γ when compared to the control group **(Fig 2B)**. Curiously, five of them were diagnosed with long-COVID-19. Already **Fig 2C** shows the status of cellular response for all subjects based on our proposed *COVID-19-cellular immunity test* concerning serology. Interestingly, COVID-19 patients showing negative cellular immune response (red solid circles; 06/26) have presented high anti-S protein IgG titers.

This initial set of results shows that the *COVID-19-cellular immunity test* was able to identify individuals with different degrees of exposition to SARS-CoV-2 and revealed COVID-19 cases in the subacute phase showing positive serology or those with an absent cellular response based on the IFN-γ-SARS-CoV-2-specific levels.

### Profile of cytokine production in response to SARS-CoV-2 Spike protein

**Fig 3:**
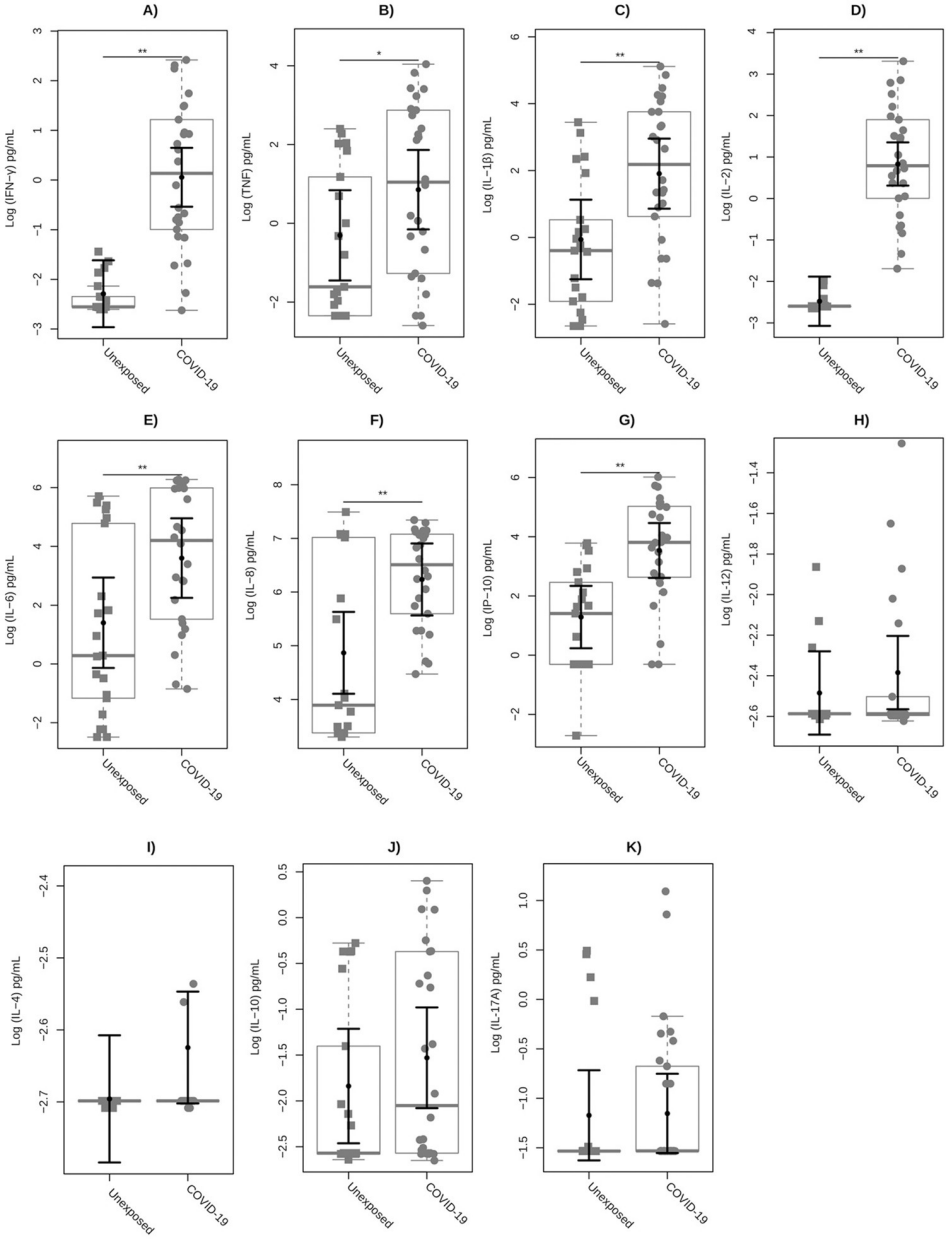
Plasma cytokine profile by SARS-CoV-2 Spike protein stimulation in whole blood CRA test. The cytokines **(A)** IFN-γ, **(B)** TNF, **(C)** IL-1β, **(D)** IL-2, **(E)** IL-6, **(F)** IL-8, **(G)** IP-10, **(H)** IL-12, **(I)** IL-4, **(J)** IL-10, and **(K)** IL-17A were evaluated. The levels obtained from each inflammatory mediator were analyzed on a logarithmic transformed scale and illustrated using box plots to compare unexposed and COVID-19 groups. Squares represent individuals in the unexposed group, solid circles represent individuals in the COVID-19 group, and the box plots represent the interquartile range and the sample median (solid gray centerline). The larger black solid circles and central bars represent the fitted means estimated by the linear model, and the marginal mean values and their upper 95% confidence intervals were estimated for both unexposed individuals and for COVID-19 controlled for sex, BMI, and age for IFN-γ and PHA (mitogen) after stimulation with a specific biomarker for SARS-CoV-2 S protein. **, P<0.01; *, P<0.05.

**Fig 4:**
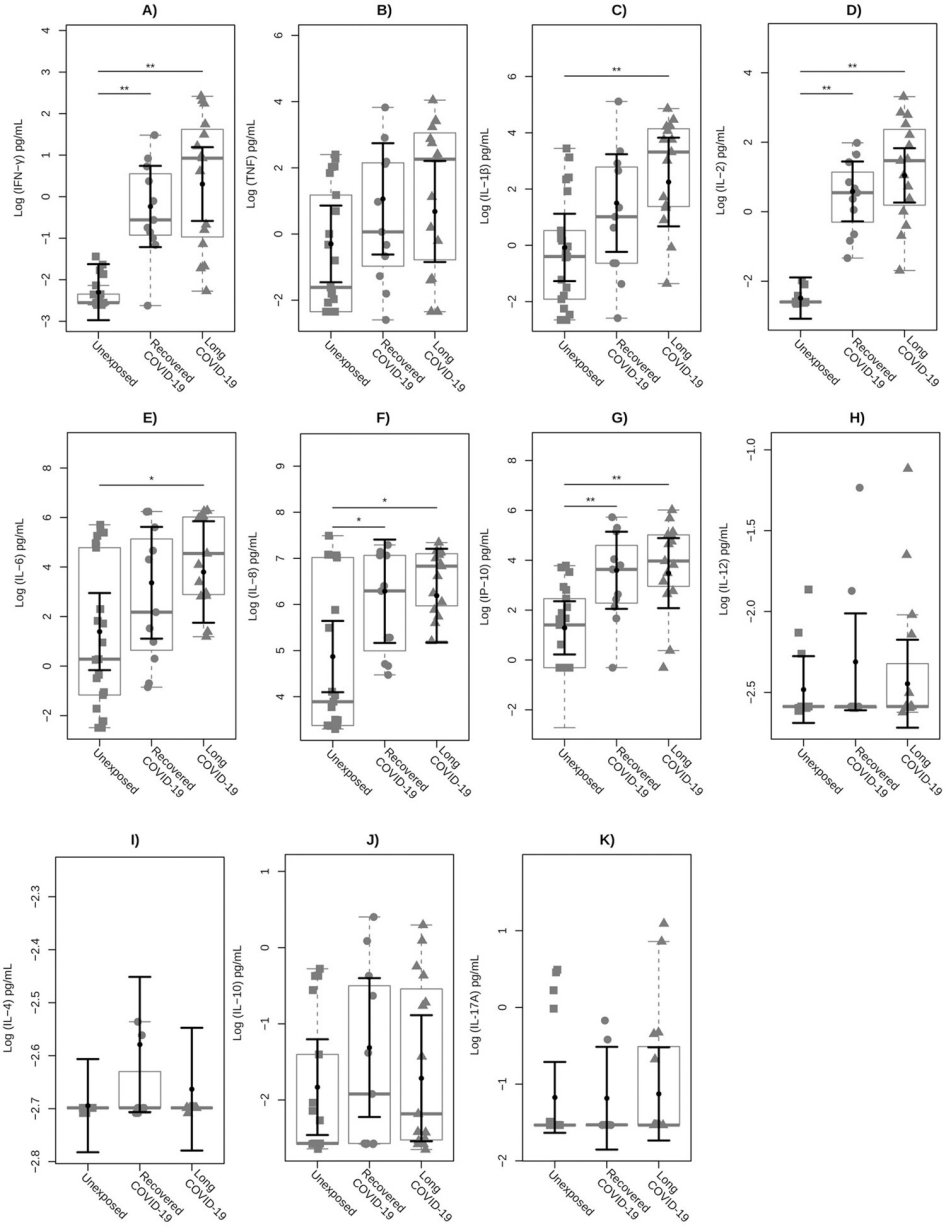
Plasma cytokine profile of the unexposed, recovered COVID-19, and long-COVID-19 groups. Cytokines **(A)** IFN-γ, **(B)** TNF, **(C)** IL-1β, **(D)** IL-2, **(E)** IL-6, **(F)** IL-8, **(G)** IP-10, **(H)** IL-12, **(I)** IL-4, **(J)** IL-10, and **(K)** IL-17A were evaluated. The obtained levels of each inflammatory mediator were analyzed on a logarithmic transformed scale and illustrated using box plots to compare the unexposed, recovered, and long-COVID-19 groups. Squares represent individuals from the unexposed group, solid circles represent individuals from the Recovered COVID-19 subgroup, triangles represent individuals from the Long-COVID-19 subgroup, and the box plots represent the interquartile range and the sample median (solid gray centerline). The larger black solid circles and central bars represent the fitted means estimated by the linear model, and the marginal mean values and their upper 95% confidence intervals were estimated for both unexposed individuals and for COVID-19 controlled for sex, BMI, and age for IFN-γ and PHA (mitogen) after stimulation with a specific biomarker for SARS-CoV-2 S protein. **, P<0.01; *, P<0.05.

Since several inflammatory mediators are involved in the immunopathology of COVID-19, a set of cytokines was evaluated by multiplex immunoassay on whole blood supernatants stimulated with recombinant SARS-CoV-2 S protein. As shown in **Fig 3**, the COVID-19 group had significantly higher levels of IFN-γ (P<0.01), IL-2 (P<0.01), IP-10 (P<0.01), IL-1β (P = 0.001), IL-6 (P = 0.002), IL-8 (P = 0.003), and TNF (P = 0.014) when compared to the unexposed healthy group. The cytokines IL-12 (P = 0.746), IL-4 (P = 0.133), IL-10 (0.155), and IL-17A (P = 0.288) did not present significant values between these groups. When COVID-19 patients were categorized into recovered versus long-COVID-19 subgroups **(Fig 4)**, we observed that IFN-γ (P<0.01), IP-10 (P<0.01), IL-8 (P<0.05), and IL-2 (P<0.01) S protein-specific levels were significantly higher in both subgroups compared to unexposed individuals. Interestingly, only inflammatory IL-1β (P = 0.008) and IL-6 (P<0.05) showed significantly increased levels in long-COVID-19 compared to unexposed healthy individuals.

Additionally, IL-2 and IFN-γ-S protein-specific levels in whole blood were significantly correlated with the anti-S protein IgG levels (rho = 0.815 and rho = 0.774; P<0.001, respectively). Other biomarkers in cytokine release assay also showed a significant correlation with the humoral response, such as IL-1β, IL-6, IL-8, and IP-10 **(S1 Table)**. Furthermore, IFN-γ measured by ELISA and CBA assay shows a strong correlation (rho = 0.91; P < 0.0001).

### Principal components analysis (PCA) in COVID-19 cytokine release assay

**Fig 5:**
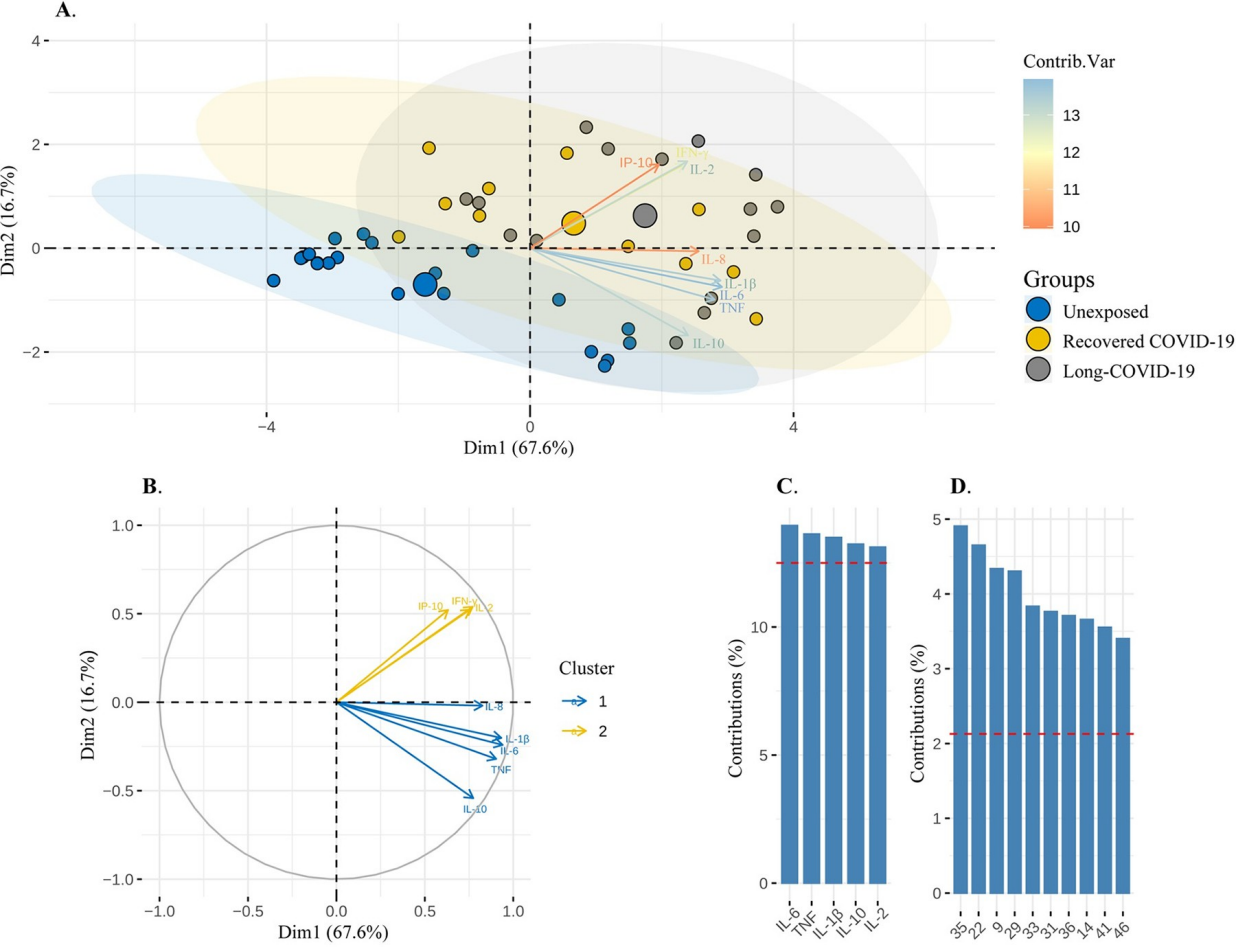
Principal component analysis (PCA) discriminates cytokines between COVID-19 and healthy unexposed groups. Analysis of variance of cytokine concentrations IFN-γ, TNF, IL-1β, IL-2, IL-6, IL-8, IP-10, IL-12, IL-4, IL-10, and IL-17A were evaluated for COVID-19 patients (N = 26) and unexposed healthy volunteers (N = 21). **(A)** A 2D representation, given by the first two principal components with 67.6% and 16.7% explained variance (84.3% cumulative percentage explained), of Unexposed (blue solid circles), Recovered COVID-19 (yellow solid circles) and Long COVID-19(gray solid circles), with point sizes proportional to the average individual contribution to any principal component. The variables (biomarkers) are expressed by colored vectors, indicating their average contribution to the principal components. **(B)** A representation where the vector represents the correlation between a variable (biomarker) and a principal component (PC) is used as the coordinates of the variable in the PC. The variables are colored according to the results of a divisive clustering of k-means (k = 2). **(C)** Bar graph indicating the seven variables (biomarkers) according to their average contribution to each main component. **(D)** Bar graph indicating the top 10 individuals according to their average contribution to any major component.

To examine whether the differential cytokine SARS-CoV-2 S protein-specific production could provide a pattern of biomarkers able to discriminate among the study groups, a PCA analysis was performed. Our results revealed that 84.3% of the total variance in response to eight biomarkers was expressed by two principal components. The first component (DIM1) represented a total of 67.6%, while the second (DIM2) represented 16.7% of the total variance **(Table 2)**. Altogether, eight biomarkers stood out as the main inflammatory mediators with discriminative potential between the COVID-19 (recovered/long-COVID-19) and healthy unexposed groups: IFN-γ, IL-10, IL-6, IL-2, IP-10, TNF, IL- 1β, and IL-8 **(Fig 5A and S1 Fig)**. Additionally, two clusters were generated according to the main inflammatory mediators; cluster 1 = IP-10, IL-2, and IFN-γ, and cluster 2 = IL-8, IL-1β, IL-6, TNF, IL-10 **(Fig 5B)**. Finally, the top-five of cytokines that most contributed to the observed variation were identified: IL-6, TNF, IL-1β, IL-10, and IL-2 **(Fig 5C)**, and an analysis of the individual average variance across the entire study population showed that the top-ten individuals who were the main contributors to the cytokine profile, six individuals had COVID-19 (recovered, N = 1; long-COVID-19, N = 5) **(Fig 5D)**.

**Table 2:** Principal-component analysis of inflammatory biomarkers in plasma from patients with COVID-19 and unexposed group.

Component	Eigenvalue	Variance (%)	Cumulative Variance (%)
Dim.1	5.4089614	67.6120180	67.6120180
Dim.2	1.3322281	16.6528522	84.2648702
Dim.3	0.6521799	8.1522493	92.4171195
Dim.4	0.2851903	3.5648789	95.9819985
Dim.5	0.1286239	1.6077994	97.5897979
Dim.6	0.0855107	1.0688845	98.6586824
Dim.7	0.0561818	0.7022729	99.3609554
Dim.8	0.0511235	0.6390445	100
Variable	Eigenvalue^&^		
	Dim1	Dim2	
IFN-γ	0.7542612	**0.5230242**	
IL-10	**0.7735441**	**-0.5420503**	
IL-6	**0.9396213**	-0.2412021	
IL-2	0.7700611	**0.5395748**	
IP-10	0.6311788	**0.5214789**	
TNF	**0.9033968**	**-0.3202663**	
IL-1β	**0.9322525**	-0.2016275	
IL-8	**0.8259485**	-0.0192687	

^&^The proportion of variation and cumulative percentage are shown for each eigenvalue.

## Discussion

The immune response plays an important role in the pathophysiology of COVID-19, being associated with the protective response as well as the long-COVID-19 severity and/or devasting complications. In this diverse scenario, a range of cytokines stands out in the orchestration of immune defense against SARS-CoV-2 infection by dictating the innate and adaptative immune system dynamics, represented by cellular and humoral responses counterparts, which are relevant before, during, and after COVID-19 [35]. In the present study, we have proposed a *COVID-19-cellular immunity test* based on the cytokine release assay (CRA) in whole blood in response to SARS-CoV-2 S protein-specific to evaluate IFN-γ levels and other cytokines and chemokines associated with different degrees of exposition and COVID-19 infection. Our data identified a significantly high production of pro-inflammatory cytokines in COVID-19 patients, even in a post-acute phase of infection, as well as in individuals who had long-COVID-19 compared to an unexposed cohort. In addition, we showed that the cytokines IFN-γ, IL-2, IP-10, IL-1β, IL-6, IL-8, and TNF could discriminate the status of SARS-CoV-2 infection or exposition, and also it highlighted the high levels of pro-inflammatory IL-1β and IL-6 in long-COVID-19 when compared to the unexposed group.

Although COVID-19 predominantly affects the lungs, it is a multisystemic and multifaceted disease resulting from an asymptomatic response or mild-to-moderate symptoms to significant sequelae presentation or even death [18–21]. Nowadays, it is well recognized a set of characteristics associated with predicting disease severity and a worse prognosis, such as arterial hypertension, obesity, and diabetes [36–40]. Our COVID-19 cohort was composed of individuals who had more symptoms in the acute phase of the disease, with frequency: fever (80.8%), anosmia (69.2%), headache (57.7%), and cough (57.7%). Also, individuals who developed long-COVID-19 reported more frequent tiredness (73.3%), cough (66.6%), and difficulty breathing (40%) two months after diagnosis. Corroborating our findings, Carfi *et al*. [41] found cough, fever, dyspnea, musculoskeletal symptoms, gastrointestinal symptoms, anosmia, and dysgeusia as the most frequent symptoms of the diagnostic phase, which is compatible with the acute phase of the disease, while the most common symptoms in the post-COVID-19 syndrome or long-COVID-19 were fatigue, dyspnea, joint pain, and chest pain. Thus, it is observed to be a pattern of symptoms more common in the acute phase, such as fever, cough, and anosmia, while some seem to be more frequently associated with long-COVID-19, such as fatigue/tiredness and dyspnea/shortness of breath, which is represented in our study population. Additionally, gender seems to influence the pathophysiological conditions triggered by COVID-19. Some research has shown that men have a higher mortality and intubation rate than women. Women, on the other hand, seem to be more predisposed to develop long-term COVID-19 and to present more symptoms in the acute phase [42–45]. However, our analysis was corrected for sex, age, and BMI variables to eliminate these biases.

The modulation of IFN signaling is well recognized as a crucial antiviral mechanism; therefore, the production or interruption of IFN release may represent a critical step in the defense against virus infection [46]. Gadotti *et al*. [5] showed that, in hospitalized patients with COVID-19, higher levels of IFN-γ were related to a worse prognosis. Furthermore, Lucas *et al*. [14] demonstrated a high correlation of IFN-γ with viral load, suggesting that the secretion of this cytokine may be increased by the amount of virus. In our cohort, the *COVID-19 Cellular Immunity Test* was based on the IFN-γ release assay (IGRA) using the recombinant SARS-CoV-2 S protein as a stimulus in whole blood, and we have shown that IFN-γ-SARS-CoV-2 S protein-specific levels were significantly higher in the COVID-19 group compared to the unexposed healthy group. Petrone *et al*. [47]found that IFN-γ levels, induced from a SARS-CoV-2 Spike mega pool, were also higher in patients with COVID-19 than in healthy controls and controls with any associated disease, demonstrating, in this case, the increase of IFN-γ was specifically associated with COVID-19. Murugesan *et al*. [48] demonstrated that in whole blood stimulation, the peptide pool to CD4+ T cells, also using the IGRA model, showed a significantly higher median IFN-γ response in convalescent individuals to healthy donors, demonstrating that the whole blood IGRA can be a useful diagnostic tool. Furthermore, in our *COVID-19-Cellular Immunity Test*, we found that in the COVID-19 group, six individuals had a negative cellular response, even though all individuals in this group had specific IgG SARS-CoV-2. Among these, only one did not belong to the long-COVID-19 subgroup. Among these five individuals with long-COVID-19, two who had chronic inflammatory diseases used corticosteroids. However, we did not observe any impact of corticosteroid therapy on our test, based on the capacity of these subjects to produce high levels of IFN-γ in response to the PHA mitogen, positive control. We hypothesize that the absence of cellular response observed in these cases could be a reflex of a dysfunctional response, which prolongs the inflammatory environment and consequently contribute to the development of long-COVID-19. The IGRA approach using a recombinant S protein from SARS-CoV-2 in a whole blood model has been shown to effectively differentiate individuals affected by COVID-19 from those not exposed to the virus, suggesting a promising assay assess the IFN-γ production by SARS-CoV-2-specific T cells in different scenarios of natural infection, vaccine or hybrid immunity. Currently, the cellular response to different COVID-19 vaccine platforms, using the assay described in the present work, is under investigation by our group.

Indeed, many studies have been conducted to characterize the cellular response in COVID-19 by exploring the SARS-CoV-2-specific T-cell response [16, 49–51]. Through stimulation with the recombinant SARS-CoV-2 S protein in whole blood, we identified a signature for COVID-19 based on the cytokines IFN-γ, IL-2, IP-10, IL-1β, IL-6, IL-8, and TNF. These cytokines showed higher levels in the SARS-CoV-2 infected group when compared to the unexposed control, suggesting a delicate balance among Th1/pro-inflammatory cytokine profile, which could be associated with a resolution outcome in COVID-19, but also a poor immune response toward long-term disease, as previously documented the Th2/Th1 imbalance associated with mortality risk [52]. Our data agree with another study that verified inflammatory profiles in COVID-19, demonstrating increases in serum levels of IL-1β, IL-6, IL-8, and TNF in patients with COVID-19 compared to healthy donors [53].

When the COVID-19 group was subdivided, only long-COVID-19 showed significantly higher IL-1β and IL-6 SARS-CoV-2 S protein-specific values compared to healthy individuals. These data suggest that these cytokines may be associated with the sequelae of COVID-19 and are supported by the meta-analysis performed by Aziz *et al*. [54], where it was seen that higher serum levels of IL-6 were found in individuals and were associated with greater severity and risk of death from the disease. Other studies demonstrate that IL-1β and IL-6 are associated with hyperinflammatory and severe disease states [53, 55, 56]. IL-6 is a pleiotropic cytokine produced by different cells, such as monocytes, macrophages, myocytes, and B and T cells, and induces the production of acute-phase proteins and adhesion molecules during infectious and inflammatory diseases [57, 58], in addition to being a crucial cytokine in the dichotomy of Th17 and induced Treg (iTreg) cell differentiation. In the presence of IL-1β and TGF-β, IL-6 leads to polarization towards the Th17 subset, which is involved in immune defense against extracellular bacterial and fungal infections [59]. Furthermore, Th17 is implicated in ARDS through neutrophil recruitment [60, 61]. Moreover, IL-6 blocks the expression of Foxp3 induced by TGF-β, impairing the development of iTreg in the periphery [62] and favoring Th17 differentiation. Therefore, the higher levels of IL-6 in long-COVID patients may decrease iTreg differentiation, contributing to an unbalanced immune response seen in these patients and perpetuating the inflamed milieu. However, one study showed that loss-of-function mutations that affect GP130, a glycoprotein that, when activated, leads to the initiation of JAK/STAT signalization, and mutations in STAT3, one of the genes encoding different components of the IL-6 signaling pathway, activating transcription, leading to cell growth and differentiation, lead to disorders such as elevated IgE and susceptibility to microbial infections in humans [63]. This demonstrates that IL-6 also has a protective role in infections. IL-1β, in turn, is an inflammatory cytokine that, in addition to mediating immune responses during infection and inflammation, has a role in chronic and acute autoinflammatory diseases [64]. It is produced after the inflammasome induces the formation of caspase-1, which cleaves pro-IL-β into IL-1β [65]. IL-1β induces the synthesis of inflammatory cytokines such as IL-6 [66]. Thus, an increase in IL-1β activity could increase IL-6 production.

Our work had some limitations, which must be mentioned, such as the relatively low number of participants, and it was performed in a pre-COVID-vaccine scenario. In addition, although the source of these cytokines has not been defined in our study, it is well-documented that IFN-γ is predominantly produced by activated CD4 T helper type-1 (Th1), CD8 cytotoxic T cells, γδ (gamma-delta) T cells, and natural killer (NK) cells, while others cytokines showed in this work could be produced by a range of leucocytes phenotypes (neutrophils, monocytes/macrophages, lymphocytes) from innate/adaptative immune system [67]. Also, we did not find modulation of other cytokines, such as IL-4, IL-10, IL-12, and IL-17A, probably due to the short evaluation time of 24h, which was defined based on IFN-γ production.

There is still a gap in the literature regarding the characterization of the inflammatory immune response in long-COVID-19. These cases represent an additional public health problem, as there is an increase in expenses with the treatment of these individuals and an increase in the morbidity of this disease, causing damage to the entire society. Some mechanisms are thought to contribute to the pathophysiology of long-COVID-19, including inflammatory damage in response to acute infection and post-critical illness-related sequelae [25]. In this sense, it is urgent to understand the pathophysiology of long-COVID-19 so that the treatment can be better targeted, whether from the point of view of the development of new drugs or the point of view of rehabilitative strategies [22]. Finally, our results showed differentiated levels of IFN-γ in response to SARS-CoV-2 infection through the *COVID-19-cellular immunity test* among exposed and unexposed individuals. We also identified a pro-inflammatory cytokine profile associated with COVID-19, highlighting the IL-6 and IL-1β, which may work as potential biomarkers or predictors of long-COVID-19 when used alone or in combination with IFN-γ. In the context of COVID-19, exploring the status of cellular immunity, which can be accessed by many elements such as activation markers, proliferation, T cell subtypes or phenotypes, or soluble mediators, may be relevant in various conditions regardless or in conjunction of humoral response over time.

## Conclusion

Our data point to important inflammatory biomarkers in COVID-19 concerning exposure and different disease outcomes, contributing to a better understanding of the pathophysiology of the disease and the development of new management strategies based on the individual’s cellular response.

## Supporting information

S1 Table Correlation between COVID-19 cellular immunity test and IgG serology. (TIF)

S1 Fig Eight biomarkers stood out as the main inflammatory mediators with discriminatory potential between COVID-19 and healthy unexposed groups. (TIF)
