# Supplementary figures and images for: High levels of pro-inflammatory SARS-CoV-2-specific biomarkers revealed by in vitro whole blood cytokine release assay (CRA) in recovered and long-COVID-19 patients
Source: PLoS One. 2023 Apr 5;18(4):e0283983. doi: 10.1371/journal.pone.0283983 (PMC10075475; doi:10.1371/journal.pone.0283983)

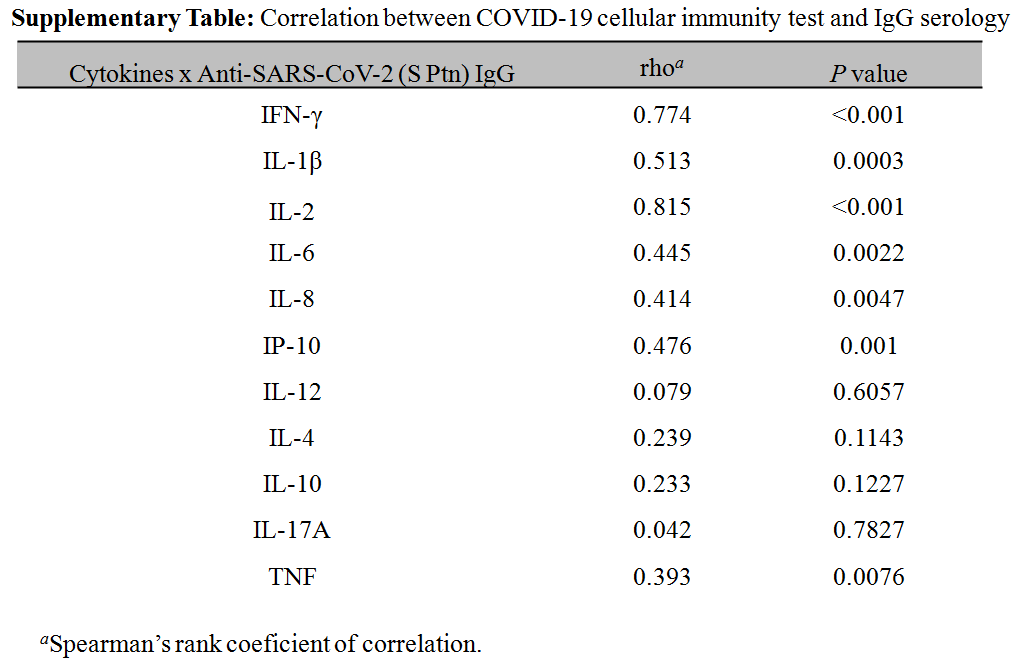

Supplement: S1 Table — (TIF) [file pone.0283983.s001.tif]

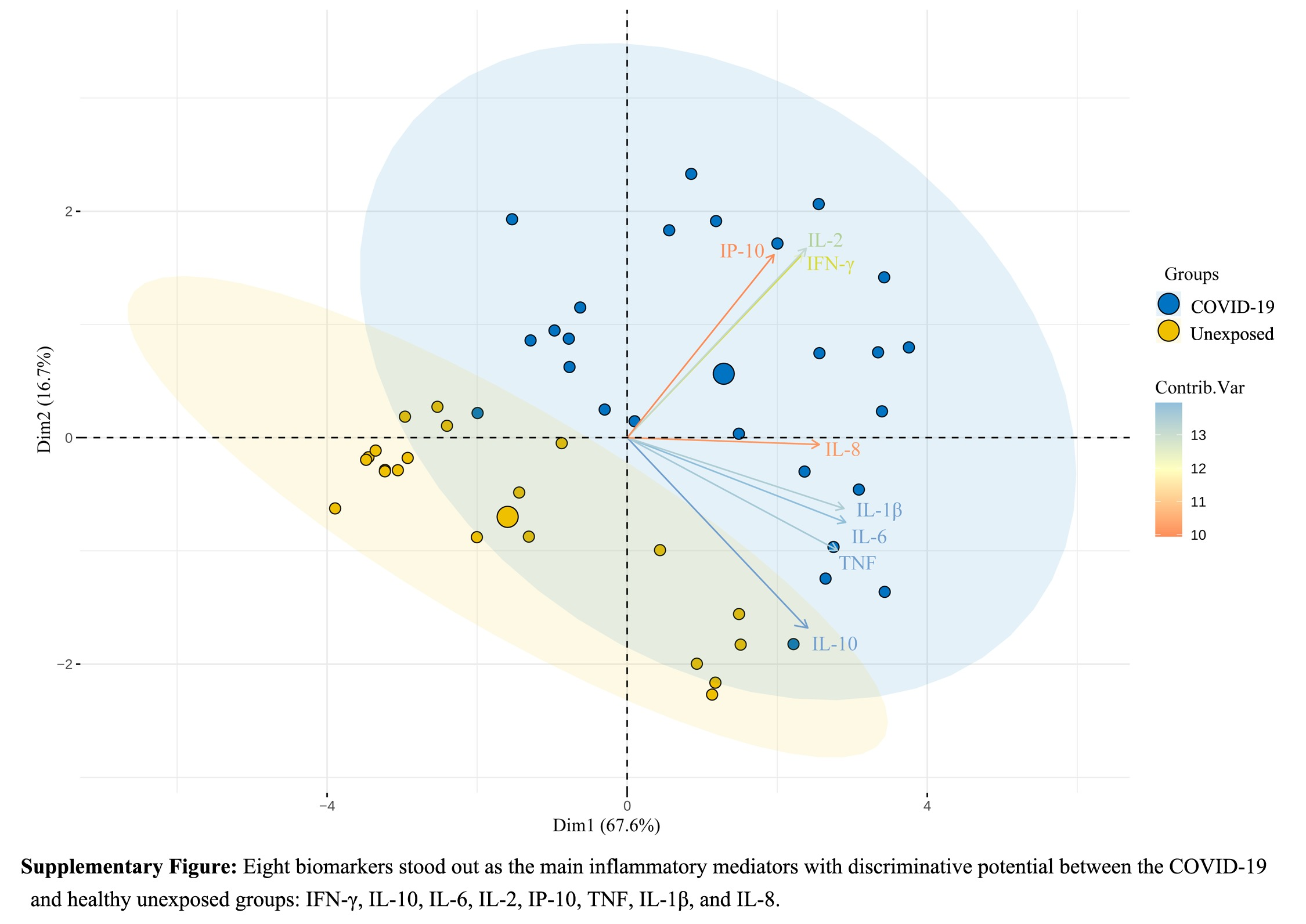

Supplement: S1 Fig — (TIF) [file pone.0283983.s002.tif]
